# Innovative solutions to enhance safe and green environments for ageing well using co-design through patient and public involvement

**DOI:** 10.1186/s40900-020-00223-4

**Published:** 2020-07-29

**Authors:** Anna L. Hatton, Catherine Haslam, Sarah Bell, Joe Langley, Ryan Woolrych, Corrina Cory, James M. W. Brownjohn, Victoria A. Goodwin

**Affiliations:** 1 School of Health and Rehabilitation Sciences, The University of Queensland, Brisbane, Australia; 2 School of Psychology, The University of Queensland, Brisbane, Australia; 3 European Centre for Environment & Human Health, University of Exeter, Truro, UK; 4 Art and Design Research Centre, Sheffield Hallam University, Sheffield, UK; 5 The Urban Institute, Heriot-Watt University, Edinburgh, UK; 6 College of Engineering, Mathematics and Physical Sciences, University of Exeter, Exeter, UK; 7 College of Medicine and Health, University of Exeter, 2.05d South Cloisters, St Lukes Campus, Magdalen Road, Exeter, EX1 2LU UK

**Keywords:** Ageing, Urban design, Physical environment, Injury prevention, Social connectedness, Activity, Community participation, Patient and public involvement, Co-design

## Abstract

**Background:**

There is a need to develop innovative solutions to enhance safe and green physical environments, which optimise health, wellbeing and community participation among older adults. To develop solutions that meet the needs of a diverse ageing population, an interdisciplinary approach is needed. Our aim was to identify the needs of older people in relation to ageing well in the environment by bringing together knowledge from different perspectives using Patient and Public Involvement.

**Methods:**

An international consortium (Retrofit living For ageing well through Understanding and Redesign of Built environments consortium: ReFURB) was established in April 2018, including ten core members, to (i) explore cutting-edge solutions to safe living for ageing populations and (ii) develop innovative approaches to everyday physical environments, which bring about health benefits. We used a co-design, interdisciplinary framework involving older adults, carers, physiotherapists, geriatricians, engineers, human movement experts, geographers and psychologists from the UK and Australia. This engaged people in a 1 day workshop that comprised a series of presentations from international speakers on urban design, social connectedness, hazards and injury prevention, and the physical environment. Small group discussions (facilitated by consortium members) followed presentations to consider the opportunities, challenges and barriers encountered with ageing, which included the use of creative engagement activities (LEGO® Serious Play, mind maps, poster gallery walk), to help participants share personal stories and reflect on the issues raised. Thematic coding was used to synthesise the outputs of the small group work.

**Results:**

Five themes were identified across the workshops: access and transport; involvement of the whole community; restoration rather than redesign; assistive and digital technology; and intergenerational approaches. These dimensions related to the physical, social and nature-based qualities of everyday environments, as they pertain to ageing well.

**Conclusions:**

Co-design was a valuable tool that helped understand the perceptions of participants and essential to develop effective interventions and solutions. Participants highlighted several issues affecting people as they age and key environmental considerations to promote wellbeing, activity, and participation. The consortium identified gaps in the existing evidence base and are now planning activities to further develop research ideas in collaboration with our co-design participants.

## Plain English summary

Our everyday physical environments can pose challenges to maintaining a healthy and active lifestyle in later life. New solutions to improve the design, safety, and accessibility of diverse environments are needed to optimise health, wellbeing, activity, and community participation in older adults. Ten academics from two UK and Australian universities with expertise in these areas came together to develop novel solutions to enhance opportunities for safe, healthy living in old age. The group held a 1 day workshop for scholars from a range of disciplines, industry representatives, patients and older members of the public. The workshop began with presentations about urban design, social connectedness, injury prevention, and the natural environment. Participants were then involved in identifying research priorities within these topics, by sharing their ideas during small group discussions. Creative activities involving LEGO®, mind maps and posters, were used to help participants share their personal experiences and provide feedback on issues raised in the talks. Together, the researchers and participants identified challenges to ‘ageing well’ and provided recommendations for possible solutions. Five main themes were identified, including access and transport; involvement of the whole community; restoration rather than redesign; assistive and digital technology; and intergenerational approaches. Older adults’ needs related to qualities of physical, social and natural environments. Feedback from participants during the workshop helped the group identify new challenges and solutions, which had not previously been considered. Findings from this work have informed the group’s future research activities, which will include collaboration and co-design with patients and the public.

## Background

Over a decade since the World Health Organization’s recommendation for ‘age-friendly cities’ [1], the built environment continues to pose major hazards (e.g. falls due to uneven terrain) to older adults [2], which contribute to reduced community participation. Characteristics of the outdoor environment — such as uneven and wet surfaces, hazards on pavements and kerbs — have been identified as risk factors for falls in middle-aged and older adults [3]. Physical inactivity is also a major public health concern, with older adults identified as one of the most sedentary age groups [4], heightening their risk of mortality, metabolic disease and obesity. Emerging evidence suggests that outdoor physical environments pose significant challenges across the adult lifespan, and may not be fit for purpose to support active and independent living into later life [5, 6].

Redesign of the built environment, informed by new knowledge of how older adults engage with their surroundings, is attracting growing attention as a means through which to improve health and wellbeing in old age [6]. Worldwide, new urban design initiatives are emerging to transform public spaces, increase physical activity, participation, and social interaction, and reduce the growing burden of chronic diseases, in older adults. Notable developments include exercise parks designed for older people’s needs [7, 8], smart home technology [9], dementia villages [10], and scoping of ‘walkable’ neighbourhood design features [11]. Sales et al. [8] reported that short-term use of a novel exercise park improved physical function in older adults, and was associated with high rates of adherence and participation. However, safety aspects of purpose-built outdoor exercise areas — for example where there is limited control over climatic and environmental changes — require further consideration [7]. Other groups have reported that the immediate physical environment surrounding an older person’s home, and opportunities to participate in exercise within a social setting, are most important to support physical activity participation [12].

Related to this, social connectedness is a major factor underpinning older adults’ community participation, and therefore, environments designed to encourage interaction with others may be effective in supporting healthy, active ageing. Several studies have explored the concept of ‘active transport’ and evaluated the key features of ‘walkable’ and pedestrian-friendly neighbourhoods, as a potential route by which to encourage physical activity in older adults [11, 13, 14]. Street connectivity, mixed land use [15], ease of access to destinations and transport and shared routes for walking and cycling are critical factors to consider in urban planning in order to help increase pedestrian activity at concentrated areas (e.g. shopping centres) which are meaningful to, and frequently visited by, older people [11]. As important as utilisation of those spaces is people’s sense of connectedness, identification and belonging to the community within which those spaces exist (i.e., their neighbourhood or community identity). Such community belonging — reflected in people’s sense of social identification as *us* Glaswegians or *us* East Enders, for example — enables access to a range of psychological resources (support, control, resilience, self-esteem, etc.) that have recognised benefits for health and well-being [16]. Particularly relevant to the present context is the positive role that such identification plays in supporting mobility [17]. Thus, finding an optimal balance in land mix, specifically urban infrastructure and green space (such as parks, gardens, woodlands and green trails), and community identification is critical. Overload of environmental stimulation, including neighbourhood retail destinations and high urban density, may negatively impact on psychological wellbeing and cognition in later life [18, 19].

Connecting with nature within and beyond our built environments, including green and blue spaces, can also play a major role in promoting healthy ageing [20], with reported mental health benefits for middle-aged and older adults [21–24]. Evidence suggests that people who live in the ‘greenest’ communities are at lowest risk of presenting with psychological distress, and tend to adopt more physically active lifestyles, allowing them to experience nearby nature [21]. Novel strategies have been implemented to facilitate interaction with green spaces including community walking maps [25] and social walking groups [26], to counter sedentary behaviour and help older adults maximise the health benefits through encounters with these surroundings and social interactions. Having a greater abundance of parks, located within close proximity to older people’s homes may be one solution to increase physical activity [27]. However, overcoming challenges in maintaining parks that are safe, accessible, clean, attractive and nuisance-free, may shape how public green spaces are utilised [6]. For older adults with a cognitive impairment, such as dementia, sensory gardens may offer therapeutic effects on psychological wellbeing and behaviour, if well thought through and carefully designed [28]. Similarly, blue spaces (e.g. lakes, oceans, coastal regions) may also contribute to therapeutic landscape experiences, which can promote wellbeing amongst older people [29]. Participation in water-based activities such as surfing [23], or simply exposure to a visible blue space [30], have been shown to reduce psychological distress, specifically anxiety and depression, in middle-aged and older adults.

Whilst evidence suggests there are multiple factors that play a major role in ‘healthy and active ageing’, it is not clear which factors are considered most important by older people. The thoughts and personal experiences of diverse older people are critical to inform the development of research agendas and projects that address the current and future needs of ageing populations. Without knowledge of varied older people’s priorities, concerning their interaction with everyday outdoor environments, it is unlikely that any solutions (exclusively designed by researchers) to enhance ‘ageing well’ would be effective or attract engagement by older people. Our aim was to explore the potential needs of older people in relation to ageing well in the environment by bringing together knowledge from different stakeholder and interdisciplinary perspectives.

To undertake this and wider research, the National Institute for Health Research (NIHR) recommends that patients and members of the public should be involved in identifying and prioritising topics, and in designing, monitoring and evaluating their outcomes [31]. An active partnership with patients and the public, initiated at an early stage in the research process can provide researchers with critical insight of lived personal experiences (e.g. what it is like to be an older person), which are used to inform the development and implementation of health research that is more relevant to the end-user. Patient and Public Involvement (PPI) can therefore be highly beneficial in developing, clarifying and affirming the importance of meaningful research questions, prior to seeking research funding. In this paper we report how PPI (referring to diverse members of the public, carers and people who use health and social care services, as well as people from organisations that represent service-users) helped to identify the research priorities for an international consortium, whose objective is to develop cutting-edge solutions to enhance safe, green everyday environments for ageing well. The concept of ‘ageing well’ refers to an individual’s vision on what *they* perceive constitutes well-being as they age, and this encompasses multiple dimensions, such as being in good physical health, having a sense of purpose, feeling socially connected and having a sense of belonging in the community [32]. PPI participants were specifically involved in the ‘Identifying’, ‘Prioritising’ and ‘Design’ stages of the research process, as a means to help plan the consortium’s future work. Creative engagement activities were implemented during the PPI process to facilitate communication of tacit knowledge and shared understanding. Our use of creative activities, over other forms of engagement activities (e.g. involvement in steering committees), seeks to address power and epistemic injustice, where the knowledge (and voices) of non-academics are typically underprivileged in such ‘co-design’ settings. Creative methods can assist in rebalancing this injustice by offering an ‘alternative’ mode of sharing and communicating that is familiar to all, yet uncommon in research settings, which typically use written and spoken ‘jargon filled’ words in ways that are often exclusive [33].

## Methods

### Conception of an international consortium

In April 2018, an international consortium of academics from The University of Queensland (Australia) and University of Exeter (United Kingdom) was co-founded by VG and ALH aiming to: (i) explore cutting-edge solutions to safe living for ageing populations and (ii) develop innovative approaches to building infrastructure, which bring about health benefits. This initiative sought to converge fundamental principles from the fields of engineering, injury biomechanics, human movement, healthcare and psychology, through the conception of the ‘Retrofit living For ageing well through Understanding and Redesign of Built environments (ReFURB)’ consortium, as a means to create and evaluate (proof-of-concept) solutions that consider both safety and active ageing. The consortium was supported by an institutional research grant (jointly awarded by The University of Queensland and the University of Exeter). Ten core consortium members (five academics from The University of Queensland and five academics from the University of Exeter) were selected for their complementary interdisciplinary expertise within the broad field of ‘healthy ageing’. The proposed consortium received positive feedback from the prospective core members, which provided affirmation of the need for international collaborative leadership to help develop new strategies to promote healthy and active ageing, and enhance safe and accessible community living and participation in later life. All consortium members were supportive of hosting a UK-based workshop, as a route through which to use public and patient engagement to inform the development of the consortium’s terms of reference, objectives, future research priorities and agenda. The workshop was held over 3 days in June 2018 and hosted at the University of Exeter.

### Workshop planning and facilitator training (day 1)

Day one involved five consortium members (including ALH, VG, CH, CC) and one of the external speakers (RW) meeting face-to-face to refine the format and content of planned activities for the following day and receive training in facilitating small groups to ensure consistency and active engagement of all participants. Several methods of participant engagement were considered including the use of LEGO® Serious Play (LSP), goal-directed personas, and mind maps. The training was facilitated by an expert in co-design and LSP (JL).

The planning meeting included facilitator training in several creative engagement activities, specifically LSP, goal-directed personas, and mind maps. Upon using LSP for the first time, the group were instructed in how to conduct ‘creative warm-up exercises’ for familiarisation purposes. This included building a model that represented a concept, to which all could relate (e.g. your home town). LSP methods were then used in a similar manner, to consider ‘challenges to ageing well’. The group also experimented with the use of goal-directed personas, whereby each person created a persona using a male or female body silhouette that represented themselves as an older adult (20+ years in the future). This model was used to help the group reflect on the perceived physical, psychological, environmental or social relational needs of older adults. However, given that workshop participants represented a breadth of ages across the lifespan (and so older delegates would need to create personas that captured their current self, rather than future needs), it was agreed that LSP would be a more widely accessible engagement tool. Our decision to implement LSP was rooted in evidence that such creative activities can be used as a means to overcome barriers between workshop participants and their experience (e.g. on the basis of status or knowledge), and to create equality of voice among a diverse group [34]. We also used participant-generated mind maps to create a graphical “visual expression of meaning”, which can facilitate clear articulation of experiences [35].

### Stakeholder engagement workshop (day 2)

Day two was the main co-design event that involved a wide range of participants. Workshop participants were recruited by invitation only, to ensure attendance from individuals with complementary expertise, from across a range of disciplines, professional and public sectors. The delegate list was compiled by two consortium members (VG, ALH) with input from the wider group, and included 44 individuals or organisations who received an invitation to attend the workshop (or identify a relevant person) via email. PPI representatives were identified through the local University of the Third Age and the Peninsula Patient Involvement Group. We aimed to recruit 40 participants, who could be divided into four groups of ten for each of the small group discussions. Refreshments were provided and members of the public who attended the workshop were reimbursed for their time and travel expenses, in accordance with INVOLVE guidelines (a UK government funded programme which supports active public involvement in National Health Service, public health and social care research) [36]. Following INVOLVE guidelines, ethical approval was not required to involve participants in the planning stage of our research (e.g. identifying and prioritising research priorities) [37].

The workshop was opened by a keynote presentation on Active Ageing given by a senior figure from Age UK (the UK’s largest charity for older people). This was followed by four key themed presentations, to which the subsequent small group discussions would be anchored:

- Theme 1) Urban design and active ageing;
- Theme 2) Built environment: hazards and injury prevention;
- Theme 3) Natural and sensory environments;
- Theme 4) Social identity and connectedness.

The facilitated small group discussions allowed participants to share their perspectives on concepts presented within each themed talk. The small groups each had two facilitators – one being the speaker who delivered the talk linked to the theme, and the second a ReFURB consortium member, with at least one having attended the facilitator training session. The discussions commenced with the facilitators clarifying the relevant workshop theme, for example, providing an interpretation of what is meant by the term ‘urban design’ or ‘social connectedness’ and asking participants how they interpreted these terms, in light of their personal experiences. Within each group, the following initial question was posed; “What are the challenges or barriers [for example, concerning the built environment]?” Using LEGO®, participants were then asked to build a representation of one challenge they had identified, and to write their idea on an accompanying label. Once all participants had constructed their LEGO® model, each member of the group described the challenge, and explained what different elements of the model signified (e.g. layered LEGO® blocks representing uneven terrain). By inviting each participant to present their own LEGO® model to the group, this enabled all voices and experiences to be heard at this stage in the process. Extended discussion of the LEGO® models was facilitated using the following standard questions across all the small groups: “Can the challenges be grouped or joined?”; “Where are the similarities in the challenges?” and; “What do you think will be the greatest challenge in the future?” Where possible, the facilitators encouraged participants to share practical examples, for example, “loss of memory” was considered a generic idea, whilst “not being able to remember my grandchildren’s names” provided greater specificity.

Following the LEGO® design activity, each group was asked to collaboratively create a mind map detailing the perceived ‘needs’ and main issues within the theme. Ideas and thoughts captured on the mind maps remained anonymous. To engage all participants in the discussion of the four workshop themes (rather than just the theme to which they had self-assigned), a 30-min poster gallery walk was conducted, during which the mind maps created by all four small groups were displayed. Participants were encouraged to add their thoughts and ideas to the posters, and to place a mark next to existing comments with which they strongly agreed. One of the small group facilitators remained at each poster to address any questions and facilitate further participant discussion. At the end of the poster gallery walk, the original participants of each small group reconvened to consider the ideas/responses from other participants, and to generate a priority list, identifying the ‘top four’ issues to be addressed within their theme. Photographs of the LEGO® models and posters were taken to aide subsequent data analysis and inform the ReFURB Consortium’s research agenda, e.g. post-workshop scoping activities.

The workshop concluded with a panel discussion, whereby the facilitators of the small group discussion provided an overview of the initial ideas posed by the keynote speakers, and new ideas that had subsequently emerged following participant engagement, specifically noting any changes in priorities based on participants’ insights and personal experiences. There was a further opportunity for any final remarks from participants.

The workshop was not audio or video recorded. Communication teams from the ReFURB consortium members’ institutions (The University of Queensland, Australia and the University of Exeter, UK) were engaged to share the workshop activities through social media outlets (e.g. Twitter). An artist also attended the workshop to create a visual summary (infographic) of the talks and small group discussions that would be used for dissemination purposes. At the outset of the workshop, participants were notified that they would remain anonymous, and any potential ideas generated during the workshops would not be disclosed for the protection of intellectual property. Any social media posts or comments would only refer to the ‘processes’ of idea generation.

### Analysis, reflection, and future planning

Day three involved a post-workshop meeting with six ReFURB consortium members (including VG, ALH, CC, JB). The purpose of this meeting was to discuss feedback, and any new ideas, that had emerged during the engagement activities, and to plan the consortium’s short-term research goals. During the workshop, ReFURB consortium members sought to identify and synthesise key ideas that arose over the course of the day – this process included making written notes of recurring themes shared by participants, and highlighting ideas that were in agreement or conflict with the groups’ original priorities. Following the post-workshop meeting, two ReFURB members (VG, ALH) thematically coded data captured in the mind maps that were created by participants during the small group discussions (Table 1), along with any other written materials collated throughout the day.

**Table 1:** Participants’ feedback on the workshop themes captured on mind maps during the small group discussions

Theme	Subtheme	Participant Feedback
**Urban design and active ageing**	*Community connectedness*	• Need for a centre / hub-point for communities to come together
		• Groups that are open and inclusive of all ages
		• Connection and communication, ensuring we can ‘connect’ people (e.g. using network technologies)
		• Inter-generational spaces
	*Transport*	• Strategies to help mobilise people “getting there”
		• Shared transport, volunteers (door-to-door service)
		• Location of transport needs
		• Social isolation can be seasonal
		• Need for safe and secure transport
	*Housing*	• Spaces that are not ‘overdesigned’, e.g. to accommodate pets with the home environment
		• Spaces between the home and neighbourhood can be the most challenging to navigate
		• Housing which connects people to the community – e.g. ‘downsizing’ through shared room schemes
	*Public spaces*	• Outdoor/indoor spaces that co-exist
		• Maintenance of public spaces, e.g. uneven pavements, curbs, location and signing of pedestrian crossings
		• Shared spaces (e.g. cyclists and pedestrians) can be dangerous. Need for segregated spaces that prioritise pedestrians
		• Green spaces for visual benefits, with strategically located and quality resting points (e.g. seating)
**Social identity and connectedness**	*Self*	• Loneliness as a core theme with multifactorial causes
		• Transitions (e.g. retirement, geographical re-location) can have physical, social, psychological implications
		• Isolation (not to be confused with loneliness), including digital isolation
		• Fragmentation in social networks
	*Others*	• Hard to reach groups – need to understand who these groups are, why are they hard to reach, what do they require to reach out and, what can be done to reach in to them?
		• Need for communal / shared living, offering benefits, insight and altered perceptions of life
		• Negative perceptions towards dependence – social drive that to be independent is seen as ‘successful’
		• Scope for a ‘Social Prescriber’ or ‘Community Builder’
	*Skills*	• Communication skills and confidence levels as factors associated with loneliness
		• Loss of social responsibility and sense of purpose – caring, time, skills
		• Optimal mobility, communication and confidence can lead to less hardship and increased quality of life
**Built environment: hazards and injury prevention**	*Physical environment*	• Hazards can include escalators, slopes, stairs, curbs, level changes, pot holes, paving slabs, limited space, poor equipment and maintenance
	*Personal characteristics*	• Fear of falling and perceived unsteadiness
		• Frailty in later life
		• Psychological changes including dementia, anxiety and mental wellbeing
		• Physiological changes including altered vision, reduced muscle strength and limited joint movement
	*Strategies*	• Adaptation to the built environment considered the most important aspect
		• Impact (e.g. when falling) leading to injury – need for absorbing surfacing
		• Lack of assistive technologies to provide safety prompts (e.g. visual, auditory cues)
		• Training needed to help learn how to successfully negotiate hazards
		• Poor culture of care – lack of education, knowledge and prescription of aids related to safety awareness
		• Altered sensory perceptions can influence hazard detection
		• Need for strategies that tap into physical reserves
**Natural and sensory environments**	*Environmental maintenance*	• Uneven ground – can be useful for tactile feedback, but poses a hazard with changes in weather (e.g. slip hazard when wet) and a risk of falls and injury
		• Countryside is not always well-maintained and can be challenging to negotiate
		• Exposure to the elements – e.g. breeze through an open window – can be uplifting and enlivening, but also disorientating
	*Psychological barriers*	• Some people are habituated to city/town areas and are hesitant to interact, or in-experienced, with the natural environment (‘Goldilocks Complex’)
		• Not all people get sensory pleasures from nature
	*Design*	Resting places (e.g. benches, seating) need to be in strategic locations and of good quality/design
		• Need for handrails
	*Navigation*	• Few mobile phone apps available for navigation in country / rural areas. Good phone signal is essential
		• Signage and an ability to read maps
		• Weather conditions can make navigation difficult – e.g. wind, fog, rain affects navigation, even more so for people with visual or vestibular impairments
		• Navigation can become challenging due to changing landmarks, e.g. landscape growth or vandalism
	*Blue space*	• Water can be a safety hazard where there are no barriers, stagnant water, or risk of disease
		• Difficulty sensing water depth, interpreting tides, or regulating body temperature (e.g. keeping warm)
		• Water can provide different sensory experiences – temperature change, tactile, visual and auditory feedback (e.g. ornamental running water features)
		• Misconception of the need for equipment / facilities to enter water
	*Sense and reminiscence*	• Natural environment can provide a source of sensory pleasure
		• Reminiscence of life story (‘time capsule’)
	*Social behaviour*	• Disabling responses to impairment in public as barriers to participation and engagement
	*Facilities*	• Areas for social interaction, which includes intergenerational interaction across the lifespan
		• Personal care, such as access to and availability of toilet blocks (including payment for use)

## Results

### Workshop participant recruitment

Thirty three people signed up to attend the workshop of whom 30 attended on the day (three were unable to attend due to illness or transport issues) in addition to six consortium members (Total = 36 people). Workshop attendees included six healthcare professionals, one non-governmental organisation representative, one artist, seven older members of the public and 21 academic staff from universities across the UK. Participants from within academia represented a diverse range of interdisciplinary fields including Engineering (six delegates), Psychology (one delegate), Human movement (four delegates), Environment and Human Health (one delegate); Medicine and Health (six delegates); Urban Design and Built Environment (one delegate); Research and Innovation (two delegates).

### Small group work

**Fig. 1:**
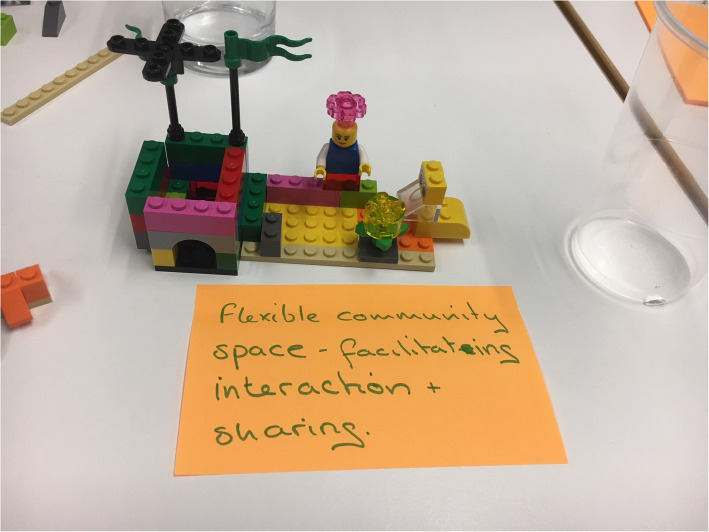
Example of a LEGO® Serious Play design innovation created by participants during the small group discussions

**Fig. 2:**
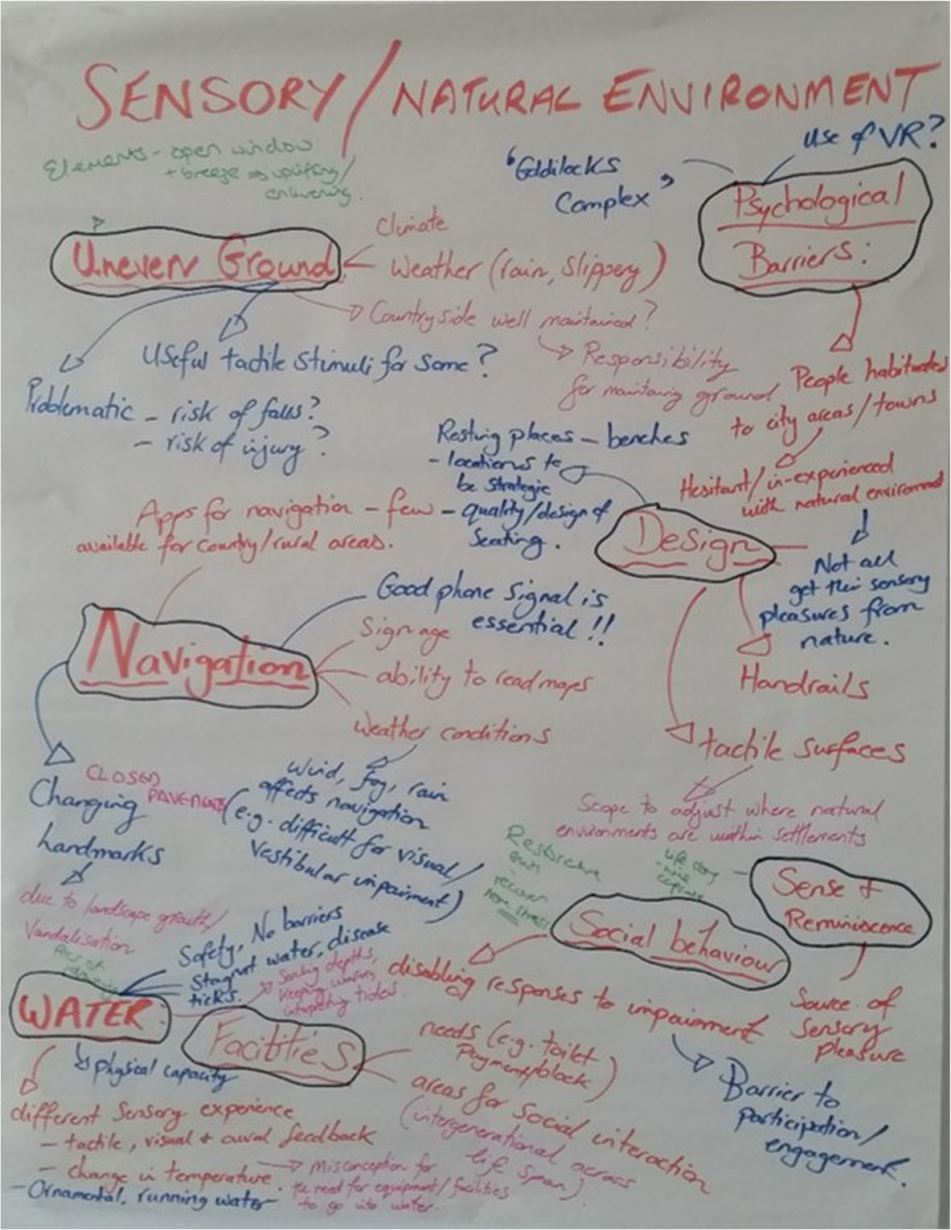
Example of a mind map capturing participants’ perceived challenges and needs within the workshop themes

During the LSP activity, workshop participants created a range of models, which represented their perceived challenges or barriers to ageing well (Fig. 1). Following this, each small group created a mind map, which pooled the ‘needs’ identified by participants within each of the four themes (Fig. 2). Table 1 summarises the main points documented within the mind maps. During the group activities, participants were asked to consider whether there were any similarities in the challenges identified, and if any challenges could be grouped together. This discussion process subsequently led to the creation of subthemes within each of the four themes (Table 1). The four main priorities, with accompanying possible solutions, identified during the small group discussions are presented in Table 2. Notably, the four priorities within each core theme represent the initial ideas from participants in each respective small group. These initial ideas were then expanded by other workshop participants during the poster gallery walk, as a means to encourage a wider collaborative approach to ideas generation. The final priorities were subsequently selected by the original small group discussion members within each core theme.

**Table 2:** Priority areas and possible solutions identified by workshop participants during the small group discussions

Theme	Priority areas and possible solutions
**Urban design and active ageing**	1. ***Connected communities:***
	• Need for a place to come together – to find out information or provide a purpose to ‘get out and about’
	• Need for physical or virtual places to connect
	2. ***Intergenerational approach:***
	• Spaces and communities which are inclusive, not exclusive
	• Simple design which accommodates all
	• Opportunities to connect generations through communication and shared learning
	• Strategies to encourage active living from early on, as past behaviour is the best predictor of future behaviour
	3. ***Getting there:***
	• Ease of access from the front door to community space, by enhancing environmental design and physical capabilities
	4. ***Sharing:***
	• Options for shared transport and housing, which include intergenerational approaches
	• Sharing of resources and skills
**Social identity and connectedness**	1. ***Loneliness:***
	• Strategies to improve confidence
	• Consideration of transitions in life (e.g. retirement), which have physical, social and psychological consequences
	2. ***Communal or shared living:***
	• Understanding what triggers a desire/need for shared versus independent living
	• Exploration of the benefits of communal living spaces
	• Need to hear insights and perceptions about shared living
	3. ***Social responsibility:***
	• Opportunities to use skills and time for others
	• Need for caring and thinking about others
	• Strategies that link people together
	4. ***Communication:***
	• Need for strategies to improve communication skills – e.g. how to find information
	• Communication considered to be a key driver related to loneliness, communal/shared living and social responsibility.
**Built environment: hazards and injury prevention**	1. ***Physical environment:***
	• Need to restore, rather than re-design, the existing built environment (most economic approach) – and consider design for the future
	• Restoration of slopes, stairs, paving, curbs, escalators, and prevention of crime
	2. ***Adaptation strategies:***
	• Need for adaptation strategies to the existing built environment
	• Potential strategies to include use of virtual reality training, education and knowledge transfer, assistive technologies (e.g. mobile phone apps, smart technologies)
	• Personal adaptation – training to tap into physical reserves
	3. ***Physical characteristics:***
	• Strategies to address physiological changes in later life, including reduced vision, joint range of motion, muscle strength, and disease symptoms (e.g. Parkinson’s disease), which impede safe interaction with the built environment
	4. ***Psychological characteristics:***
	• Strategies to address psychological changes in later life, including dementia, anxiety, fear of falling, fear of crime, wellbeing, perceived unsteadiness, which impede safe interaction with the built environment
**Natural and sensory environments**	1. ***Strategies to address psychological barriers:***
	• Introducing / increasing positive experiences with nature (e.g. use of media for sensitisation or virtual reality; facilitated repeat visits within the natural environment)
	• Understanding personal and collective barriers (e.g. fear, previous negative experiences within the natural environment)
	2. ***Restorative sensory experience:***
	• Maintenance or restoration of uneven ground to reduce risk of trips or falls
	• Increasing sensory pleasures, through sight, sound, touch (e.g. greater use of water features) but without overload
	• Increased exposure to and appreciation of microclimates (e.g. hot house, water, breeze, smell)
	3. ***Design for access:***
	• Need for more hand rails, maintained paving, accessible transport to facilitate access within the natural environment (e.g. gardens, countryside)
	• Welcoming and accessible transport stops, including community transport schemes, with upskilling of industry to effectively interact with, and support, older people.
	4. ***Facilities and inclusive events:***
	• Organised events programmes within the natural environment, with knowledgeable facilitators/staff
	• Accessible facilities (e.g. toilets)
	• Areas for social interaction, including ‘social hubs’, acknowledging the need for quiet areas

Five cross-cutting themes were identified during the group feedback session. These were: Access and transport; Involvement of others; Restoration not redesign; Assistive and digital technology; and, Intergenerational approaches. During the post-workshop meeting, further discussion highlighted additional areas to be explored based on existing evidence gaps. Several themes were interrelated, for example, ‘Access and transport’ considered how people move within environments, while ‘Assistive and digital technology’ pointed to a need for devices that provide hazard awareness (e.g. pedestrian safety). Similarly, ‘Involvement of others’ and ‘Intergenerational approaches’ shared complementary ideas, such as the role of meaningful interactions amongst individuals across the lifespan, to facilitate a sense of social connectedness, purpose and responsibility in the community. All five themes warrant further investigation in future research, but some posed particular challenges. For example, we cannot develop solutions to enhance safe access and transport of older adults, until we first identify what older pedestrian accidents are and involve (e.g. obstacles, terrain).

**Fig. 3:**
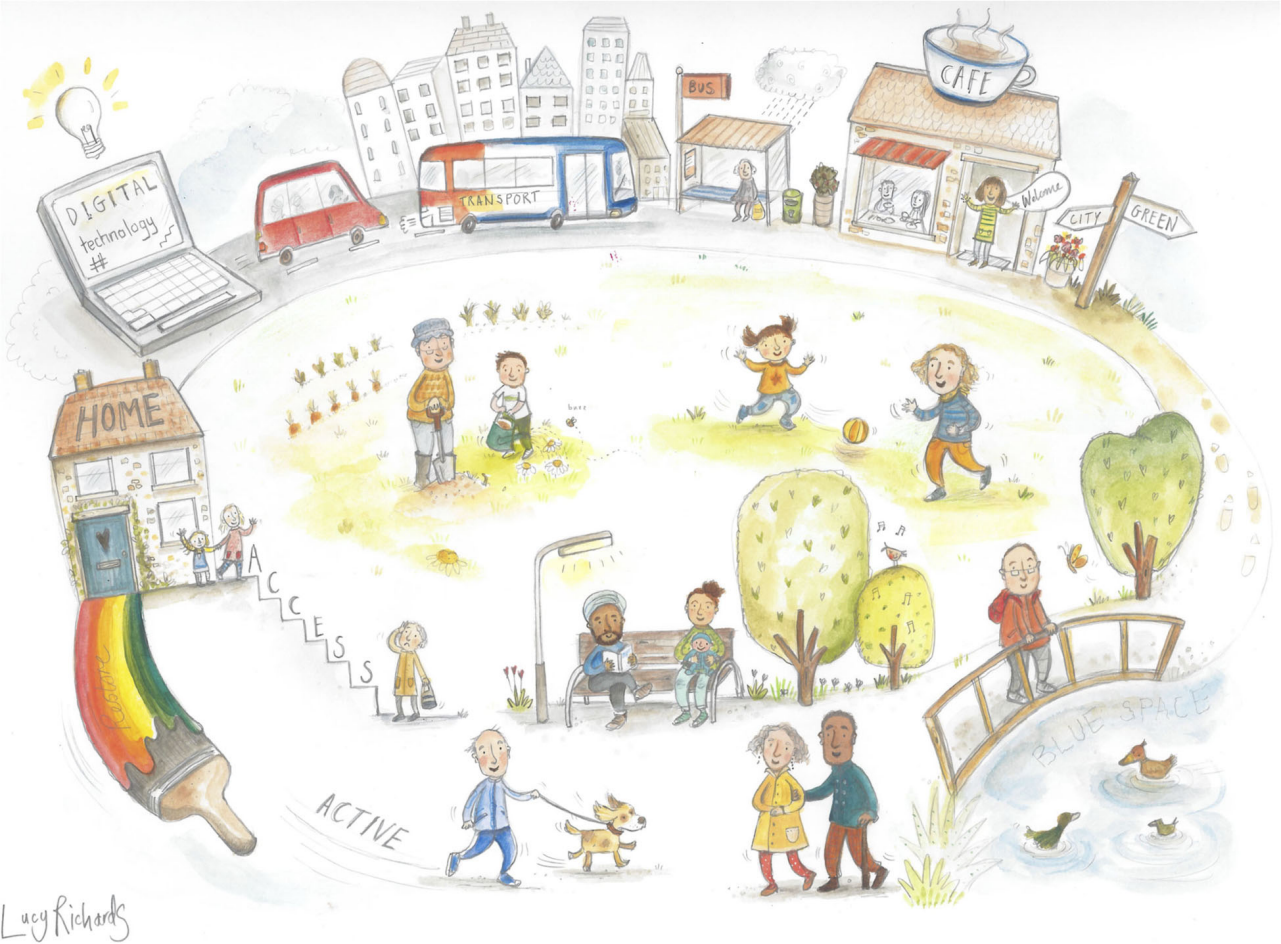
Infographic of the key themes, discussions, and feedback from the workshop

Over the course of the workshop, the artist in attendance captured ideas and discussions during the talks and small group work to produce an infographic (Fig. 3). One of the authors (VG) worked in collaboration with the artist to develop a visual representation of the day. This visual summary was used to disseminate the workshop outcomes to relevant UK and Australian organisations concerned with ageing. The workshop activities were also summarised within a blog, https://ageingrehabresearch.wordpress.com/.

## Discussion

This paper describes how we used a broad range of PPI activities to identify and develop the research priorities and agenda of a new international consortium, which was established to develop innovative solutions to enhance safe built and natural environments for ageing well. Following the conception of the ReFURB consortium, the core members organised and hosted a three-day workshop with select delegates from academia (Australia and UK), national health and government organisations, and the general public. The structured workshop programme included a series of talks, followed by facilitated small group discussions, incorporating creative engagement activities and sharing of ideas between groups. Although the day was structured around four initial themes, activities identified five cross cutting themes which should be considered when planning research and policy changes to promote active ageing.

The first cross-cutting theme and research priority identified through our PPI work was *‘Access and transport’*. At the outset of the workshop, there was a strong emphasis on how to enhance the design of specific locations (e.g. houses, parks, shopping malls), overlooking the means by which these spaces are accessed in the first instance. Participants’ feedback demonstrated a common trend pointing to the need to consider how people ‘travel to and from’, and ‘access’ diverse everyday environments, in order to interact with them. Accessible transport options impact on social inclusion, autonomy and independence [38]. Ensuring older people, including those living with illness, impairment, and disability, can remain active within and beyond their own communities requires a rethink in current transport strategies [39]. Proportionately more older people live in rural communities, when compared with their urban counterparts and this poses challenges for transport services to meet the needs of spatially unequal population distribution [40].

Whilst the consortium had acknowledged the critical role of ‘social connectedness’ within healthy and active ageing, this was largely confined to interaction with other people. Through the mind maps and small group discussions, research priorities for workshop participants indicated that the concept of connectedness should extend beyond friends and family to the ‘*Involvement of others/community’* [41–43]. It was recommended that the consortium also consider the role of, and interactions with, animals, pets, transport personnel (e.g. bus drivers who facilitate access to the environment), shared-living to address loneliness, and the need for a sense of purpose and social responsibility.

A primary aim of the consortium was to develop innovative solutions to enhance green diverse outdoor environments, for example through the creation of novel assistive devices and state-of-the-art interior design. Contrary to this assumption, outcomes of the PPI activities indicated that *‘Restoration rather than re-design’*, of such environments in the short-term was of greater importance to ageing well. There was a strong emphasis on making use of “what we already have” rather than attempting to reinvent the wheel, with recommendations that we focus on developing strategies that facilitate ‘adaptation’ to the existing environment and one which supports a sense of agency, independence and autonomy for the older adult. Large scale urban design projects often involve rapid physical transformation of city and community environments which can impact on local identity and social networks [44].

Workshop participants supported the high potential for new *‘Assistive and digital technologies’* to enhance the safety of older adults. Specific recommendations from participants, captured within the mind maps, included the development of technologies that address ‘hazard awareness’ (e.g. pedestrian safety). As well as being used for safety, assistive technology can affect where and how people live for example, driverless cars and smart homes [38]. Technology can also be used to reduce social isolation [45] and can improve care and quality of life [46], yet concerns were also identified around privacy, monitoring and digital exclusion.

Throughout the workshop talks and small discussion groups, the consortium had presented ideas that were exclusive to older adults. Whilst this approach was rooted in the overarching theme of the workshop, being ‘healthy and active ageing’, it became apparent that we should extend our focus to consider adults across the lifespan and experiences across different age cohorts. Interactions between younger and older adults were viewed by participants as being critical for physical and social wellbeing [47], and a recent review has highlighted that intergenerational contact can reduce ageism [48]. With no real understanding as to when ‘ageing’ begins [49], there is no strong rationale to limit the consortium’s remit to older adults but to have an inclusive approach that enables all to realize their potential for physical, social, and psychological well-being throughout the life course [50]. It is also important that our future work adopts an intersectional approach, which additionally considers how experiences across different genders, ethnicities, sexualities and disabilities, impact ageing well.

### Strengths and limitations

One of the strengths of our approach was using creative methods that have been shown to facilitate the articulation of tacit knowledge [51] and develop a shared understanding through co-design [52]. Creative engagement activities, as part of the PPI process, can also help to rebalance power and epistemic injustice between academics and non-academics [33]. Further strengths of this work include our interdisciplinary learning approach, which sought to bring divergent perspectives together, to create a shared vision concerning solutions to enhance ageing well.

There were, however, several limitations to this work. First, it is important to acknowledge that the PPI activities attract a self-selecting group of participants, who may not be representative of the wider population [53]. Importantly, only 7 of the 36 workshop participants were PPI representatives. As such, it is likely we did not achieve the balance in voice pertaining to the ‘lived’ (i.e. older people) versus ‘professional/academic’ experience envisioned. Among the professional and academic representatives were a proportion of older adults who were able to contribute their own experiences of ageing. Though this does not negate the importance of more actively seeking a diverse range of older non-academic participants to gain a more representative PPI voice. Our participant recruitment strategy was by invitation only, and predominantly included individuals within the consortium’s established networks; the intention being to target potential future partners (who would provide complementary expertise to that of the ‘core’ consortium members) in our initial research planning. However, our approach to participant selection may have attracted individuals who were supportive of the consortium’s work and research agenda. To address this imbalance of voice in future work, and to attract overlooked and under-represented groups, a purposive sampling approach to recruitment would be beneficial, in addition to coding of data based on ‘group representation’.

There were also limitations around workshop location and experiential data collection. While the ReFURB consortium included core members from the UK and Australia, the workshop was only delivered in the UK. Findings from the workshop may not be applicable to the diversity of older adults living in other countries, such as Australia, in light of urban, social, cultural, ethnic, and geographical differences. Third, the workshop talks and small group discussions were not audio or video recorded, in order to ensure participant anonymity and confidentiality, and protection of intellectual property. Whilst the facilitators did take notes during all sessions, and workshop participants were encouraged to document their thoughts and ideas on paper during the LEGO®, mind map and poster gallery walk activities, it is possible that some data, and importantly voices, may have been lost in the absence of recordings. Details of in-depth conversations and discussions, offering rich data concerning participants’ perceptions, may not have been captured within the abbreviated mind maps and posters. However, we felt that recording the small group discussions may have made group members more reluctant to share their views. Similarly, data collected from all small group members were collated and unidentified as they represented co-created ideas: we did not record which perceptions or ideas were from individual PPI representatives, professionals or academics. Further, following the workshop, we did not formally collect data concerning the participants’ thoughts towards the activities, or their ideas on how we could improve the co-design process. This would be a valuable approach to adopt in future work, in addition to involving PPI representatives in data analysis to re-affirm correct interpretation and meaning of data.

Finally, the workshop was designed around four a priori themes, which the consortium deemed to be important for ageing well. Whilst participants did not have the opportunity to create their own individual themes, their perspectives did contribute to the shared outcomes of the workshop. Slightly fewer people than planned attended the workshops. This was due to travel issues and illness on the day. Their absence may have resulted in new ideas or perspectives being missed.

## Conclusions

The engagement work reported in this paper provided critical insight into the personal experiences of ageing participants from different perspectives, the challenges encountered when interacting with diverse everyday environments, and possible solutions. Feedback from the workshop participants helped to inform the ReFURB consortium’s research priorities and agenda to enhance ageing well. Engaging patients and members of the public and other key stakeholders in the identification of research topics in the early stages of establishing the consortium, challenged our initial ideas and assumptions, and led to the development of research themes that were considered of greater importance to older people. Whilst the workshop participants affirmed the importance of targeting specific built and nature-based environments as a means by which to enhance healthy and active ageing, specific areas for investigation emerged, including: access and transport; the involvement of others and community; restoration of the environment rather than re-design; the development of assistive/digital technologies; and an intergenerational approach. Several workshop participants expressed their interest in continuing to help with subsequent stages of the research process (e.g. during the conception of a pilot research study aims and design), during which we intend to incorporate further stakeholder engagement and PPI.

## Data Availability

Not applicable.

## Abbreviations

LSP: LEGO® Serious Play
NIHR: National Institute for Health Research
PPI: Patient and Public Involvement
ReFURB: Retrofit living For ageing well through Understanding and Redesign of Built Environments
